# Health inequalities in hepatocellular carcinoma surveillance, diagnosis, treatment, and survival in the United Kingdom: a scoping review

**DOI:** 10.1038/s44276-025-00126-5

**Published:** 2025-03-03

**Authors:** Christopher Mysko, Stephanie Landi, Huw Purssell, A. Joy Allen, Martin Prince, Gary Lindsay, Steven Rodrigues, Jenny Irvine, Oliver Street, Deepankar Gahloth, Sara MacLennan, Karen Piper Hanley, Neil Hanley, Varinder Singh Athwal

**Affiliations:** 1 Manchester University NHS Foundation Trust, Manchester, UK; 2 University of Manchester, Manchester, UK; 3 Roche Diagnostics Limited, Welwyn Garden City, UK; 4 Vocal, Manchester, UK; 5 University of Aberdeen, Aberdeen, UK; 6 University of Birmingham, Birmingham, UK; 7 University Hospitals Birmingham NHS Foundation Trust, Birmingham, UK

## Abstract

**Background:**

Hepatocellular carcinoma (HCC) remains a deadly cancer in the UK despite advancements in curative therapies. Societal conditions and health inequalities influence the development of chronic liver disease and outcomes from complications including HCC. Scoping this emergent evidence-base is required to inform research and solutions for the NHS.

**Methods:**

A PRISMA scoping review was performed up to September 2023. Articles exploring health inequalities in HCC involving the UK population were included.

**Results:**

This review has characterised axes of health inequality and their impact across the HCC care continuum in the UK. Studies predominantly employed a cohort design or population-based analyses, with meta-analyses of surveillance utilisation including only a single UK study. These methodologies provided an appropriate lens to understand longitudinal trends and identify disadvantaged groups. However, important evidence gaps remain, including exploration of patient perspectives, intersectional analyses, and statistical measures of socioeconomic inequity in HCC.

**Conclusions:**

HCC is a rapidly growing cause of cancer mortality and disproportionally affects underserved groups, presenting a major public health concern. Further research is required to innovate and evaluate surveillance and management pathways to reduce systemic inequities. Direction is needed at the national level to improve prevention, early diagnosis and access to curative treatment.

## Background

Hepatocellular carcinoma (HCC) remains a deadly cancer in the UK despite advancements in curative therapies [1]. Due to the growing burden of chronic liver disease (CLD) in the general population, HCC is becoming one of the fastest growing causes of cancer mortality [2, 3]. Concern around this evolving public health problem has stimulated national efforts to improve standards of surveillance and management of HCC [4–7]. Social determinants and health inequalities are closely linked to the development of CLD, this extends to complications of chronic inflammation and fibrosis such as HCC [8, 9]. The World Health Organization [10]) defines health inequalities as differences in health status or in the distribution of health resources between different population groups, arising from the social conditions in which people are born, grow, live, work and age. In CLD, there is a multi-layered interaction between the aetiological causes and access to the appropriate healthcare [11]. This combines with geographical variations in socio-economic deprivation, provision of liver services and burden of disease, which all contribute to inequitable clinical outcomes [12–14]. The seminal Marmot Review [15] asserts that reducing health inequalities is a matter of fairness and social justice, and must focus on reducing the social gradient in health. A better understanding of how health inequalities interact with HCC outcomes for the UK population is urgently needed to inform future research and improvements to liver disease care pathways. Thus, striving for improved and equitable access to curative treatment for patients in line with the NHS Long Term Plan and Core20PLUS5 initiative [16, 17]. A scoping review was chosen to map this emergent body of literature and identify knowledge gaps [18]. The aim is to determine the extent of research undertaken, how well subgroups and regions have been represented, the methodologies used and whether they are sufficient in characterising health inequalities and their impact on outcomes across the HCC care continuum.

## Methods

A scoping review was conducted in line with the updated Preferred Reporting Items for Systematic reviews and Meta-analyses (PRISMA) standards, and extension for scoping reviews [19, 20]. A comprehensive database search included MedLINE, EMBASE, CINAHL and Cochrane Library to identify studies published from inception until September 2023 (when the search was conducted). A search strategy using Boolean logic and MeSH terms was developed to identify studies which focused on a population of HCC or primary liver cancer (PLC); and explored the phenomenon of interest, the impact of health inequalities on outcomes including surveillance, diagnosis, treatment, and survival. To ensure inclusivity, the search strategy was not narrowed for study type or design, therefore formal use of the PICOS or SPIDER criteria was not required [21]. No limits on time, language or type of article facilitated inclusive evidence mapping. After identification and removal of duplicates, records underwent title and abstract screening, full text for reports were then assessed for eligibility. Subsequently, citation searching was performed on all included studies to identify further relevant reports. No automation tools were used. Peer-reviewed original articles were eligible for inclusion if they involved the UK population, defined as studies where the study population included individuals from the UK, even if the population also included individuals from other countries (e.g., in meta-analyses). Articles which focused on liver disease more broadly and abstracts were excluded. An R package and Shiny app was used to produce a PRISMA 2020 compliant flow diagram [22]. Study design, cohort, setting, period, dimensions of HCC care, axes of health inequality, key findings and implications were recorded and organised in a literature matrix table to facilitate data charting and synthesis. Critical appraisal was performed for all included articles with key limitations recorded in implications in the table and an appraisal of the evidence included in the discussion. The full search strategy and eligibility criteria are included in the supplementary material.

## Results

The results section has been presented in terms of the study selection and relevant design parameters, followed by the axes of health inequality identified and their impact on clinical outcomes across the HCC care continuum.

### Study selection

**Fig. 1:**
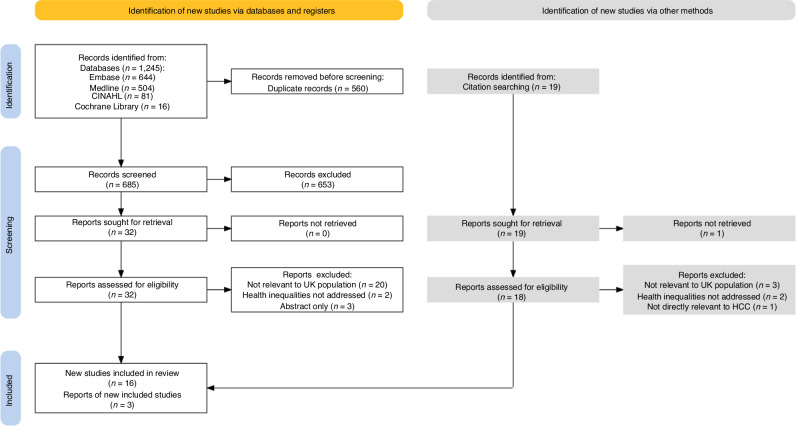
PRISMA flow diagram. Demonstrating the flow of sources through the different phases of identification, screening, and inclusion. Reasons for report exclusion after full-text assessment are included. 19 original articles were included in this scoping review. PRISMA Preferred Reporting Items for Systematic Reviews and Meta-Analyses, UK United Kingdom, HCC hepatocellular carcinoma.

This scoping review identified 1264 records, after removal of duplicates 704 records underwent title and abstract screening. The predominant reason for screening fail was unsuitability, as per the eligibility criteria. A single report was not retrievable to assess. Subsequently, 50 full reports were assessed for eligibility and 16 new studies, and 3 reports of new studies were included (Fig. 1).

### Study design parameters

The new studies (Table 1) predominantly adopted a retrospective cohort design or nationwide population-based analysis; single studies used comparative retrospective and prospective cohorts, a prospective longitudinal design, and projected future disease burden using an age-period-cohort model respectively. Cohorts of new PLC or HCC cases were obtained through regional hepato-pancreato-biliary (HPB) multi-disciplinary meeting (MDM) outcomes or from national cancer registries with linked hospital episode statistics (HES) and mortality data. A single study utilised a large primary care database [12]. The methods for identifying cohorts of patients with CLD active in HCC surveillance included screening patient records for “cirrhosis”, interrogating radiology ultrasound requests and using the Hepatitis C Research UK database linked with national cancer registry data. One study generated a cohort listed for or in receipt of liver transplantation from a national registry [23]. The setting varied from single-centre to wider regions covered by a tertiary Hepatology service, to national registries and population-based data. In the latter, different combinations of nations within the UK were represented. The period across studies included data from 1968 to 2021. The study characteristics, dimensions of HCC care, axes of health inequality, key findings, and implications of new studies are presented in Table 1.

**Table 1:** Summary of new studies addressing health inequalities in HCC in the UK, grouped by study design and ordered by year of publication (most recent first).

Study	Design	Cohort and Setting	Period	Dimensions of Care	Axes of Health Inequality	Findings	Implications
Liao et al., 2023 [12]	Retrospective cohort study	7,331 PLC cases in England from QResearch Primary Care Database	2008–2018	Diagnosis Treatment Survival	Age Deprivation level Ethnicity Sex	Age, sex, deprivation, ethnicity, and regions associated with higher incidence, late diagnosis, emergency presentation. Older age emergency presentation and lower treatment rates.	Complex public health approach recommended. Research on early detection is key.
Hamill et al., 2023 [25]	Retrospective cohort study	1,908 cases of cirrhosis with cured HCV across the UK from linked Hepatitis C Research UK resource and diagnostic imaging dataset	2012–2015	Surveillance	Accessing services Age Region	Inefficient ( > 10000 scans to treat 49 cases curatively). Low adherence 19% in first 3 years post SVR then 9% for all follow-up. Higher uptake associated with transplant centre and older age.	Surveillance poorly targeted, inefficient, and inequitable. Need for standardisation and better monitoring at national level.
Geh et al., 2022 [59]	Retrospective and prospective cohort study single centre	310 HCC cases in North East and Cumbria, England from regional HPB MDM	2019–2021	Surveillance Diagnosis Treatment Survival	Accessing services Aetiology	Covid-19 pandemic resulted in reduced surveillance adherence and HCC detection and increase in symptomatic presentation and larger tumours. Surveillance associated with better survival.	Advice resuming HCC surveillance in line with guidelines.
Beecroft et al., 2022 [14]	Retrospective nationwide cohort study	2160 HCC cases across England from national cancer registry and linked data	2016–2017	Treatment Survival	Aetiology Region	Majority (58.4%) untreated, low curative treatment (24.4%), ARLD lower treatment rates. Survival low. Variation in outcomes in regions with similar incident rates. London appears to be an outlier.	Deeper exploration of regional treatments and screening practices are needed. Lack of national data on cancer stage at diagnosis.
Farrell et al., 2017 [24]	Retrospective cohort study single centre	804 patients receiving HCC surveillance at Royal Liverpool Hospital identified from radiology requests	2009–2013	Surveillance Treatment Survival	Accessing services Aetiology	Low adherence 45%. Suboptimal performance of radiology-led recall system. Surveillance associated with improved survival.	Barriers to surveillance and discontinuation reasons need to be identified. High-quality studies of current surveillance practice required.
Selvapatt et al., 2016 [47]	Retrospective cohort study single centre	898 cases of cirrhosis eligible for surveillance at Imperial College Healthcare NHS Trust	2013–2014	Surveillance Diagnosis Treatment Survival	Accessing services Age Sex Region	Reasons for overdue surveillance presented. No significance between characteristics and adherence / adherence and outcomes.	Limited by 6-month study period. Basic patient (DNA), system and clinical reasons for non-adherence / discontinuation presented.
Haq et al., 2021 [44]	Real-world prospective longitudinal study	985 HCC cases across Glasgow and Edinburgh, Scotland from regional HPB MDMs	2009–2015	Surveillance Diagnosis Treatment Survival	Accessing services Aetiology Age Sex	60% symptomatic diagnosis. 76% adherence for those in surveillance. Younger age, females, ARLD, viral and MASLD associated with surveillance. Adherence associated with earlier stage, curative treatment, and improved survival after accounting for lead time bias.	Adherence is critical for effectiveness. Poor adherence similar outcomes to no surveillance. Pragmatic definition of adherence – ultrasound within 9 months of HCC diagnosis.
Smittenaar et al., 2016 [2]	PLC incidence and mortality projections using an age-period-cohort model with natural cubic splines	N/A	1974–2014 UK data used to estimate 2015–2035 projections	Diagnosis Survival	Age Sex	PLC projected to have the highest average annual increase of all cancers in the UK over the next 15 years. Increasing PLC mortality	Greater efforts required to tackle risk factors. Planning for increasing burden of cancer is needed. Possible artefactual increase in mortality as poor concordance with PLC diagnosis and death certificate information. Liver is common site of secondary metastases.
Burton et al., 2022 [13]	Retrospective population-based nationwide analysis	15,468 HCC cases across England from national cancer registry and linked data	2010–2016	Diagnosis Treatment Survival	Age Deprivation level Sex Region	Highest incidence in North and London. Increasing deprivation and age associated with emergency presentation and less curative treatment.	Identified regions requiring additional resources.
Burton et al., 2021 [1]	Retrospective population-based nationwide analysis	82,024 PLC cases across the UK From national cancer registries	1997–2017	Diagnosis Survival	Gender Region	Higher incidence in men. Highest incidence and mortality in Scottish men. No difference in survival between genders.	Improvements in survival for HCC, although increasing risk from obesity, diabetes and alcohol excess a public health concern.
Webb et al., 2019 [23]	National registry analysis	11,188 UK listings for liver transplant (8490 transplanted)	1995–2014	Treatment Survival	Accessing services Aetiology Age Sex Region	Increasing travel time associated with increased death after listing, and reduced likelihood of transplantation or recovery.	Inequitable accessibility of liver transplantation. Bristol is suggested as optimum site for an additional UK transplant centre to mitigate effect of travel time.
Konfortion et al., 2014 [27]	Retrospective population-based nationwide analysis	40,945 cases PLC in England from National Cancer Data Repository	1990–2009	Diagnosis	Deprivation level Sex	Increasing incidence of PLC in both sexes, largely driven by increasing HCC in men from most deprived quintile.	Rising incidence may be due to variation in known risk factors.
Ladep et al., 2014 [28]	Retrospective population-based nationwide analysis	PLC cases in England and Wales from National Cancer Data Repository	1968–2008	Diagnosis Survival	Ethnicity Sex	Rising incidence and mortality from PLC. Particularly HCC in men. Higher in migrants from high incident countries. Histology increasing mode of diagnosis.	Increasing use of histology for diagnosis may be increasing case ascertainment.
Jack et al., 2013 [29]	Retrospective population-based nationwide analysis	17,458 PLC cases in England from national cancer registry	2001–2007	Diagnosis Survival	Age Deprivation level Ethnicity Sex	Variations in the incidence and survival of PLC between ethnic groups and deprivation level. Likely due to established risk factors like HBV/HCV prevalence.	Awareness of higher risk groups. Country of birth, age at migration and length of stay in England should be recorded in future research.
West et al., 2006 [30]	Retrospective population-based nationwide analysis	PLC cases from National Cancer Registries	1971–2001	Diagnosis	Age Sex	HCC remains commonest PLC in males. Decreasing incidence in older age groups.	Ongoing burden from HCC in males. Unclear why reducing incidence in elderly.
Haworth et al., 1999 [31]	Retrospective population-based analysis of first-generation migrants	10,521 deaths from cirrhosis and 3237 deaths from PLC from mortality data in England and Wales	1988–1992	Survival	Ethnicity Sex	Higher mortality from PLC in male migrants from Asia, Africa, Scotland, and Ireland.	Prevention and screening resources could be targeted for certain ethic groups. Screening and benefit/cost ratio requires further research.

*HCC* hepatocellular carcinoma, *PLC* primary liver cancer, *HCV* hepatitis C virus, *UK* United Kingdom, *SVR* sustained virologic response, *ARLD* alcohol related liver disease. *NHS* National Health Service, *DNA* did not attend; HPB, hepato-pancreato-biliary, *MDM* multi-disciplinary meeting, *MASLD* metabolic-dysfunction associated steatotic liver disease, *HBV* hepatitis B virus.

The reports of new studies (Table 2) were all systematic reviews with meta-analysis of surveillance utilisation across studies internationally, and all included a single UK retrospective cohort study [24] within the synthesis. These were therefore deemed relevant to the UK population. However, it should be noted the included studies were predominantly conducted in the USA, with fewer studies from Europe and Asia. Included studies were predominantly retrospective or prospective cohort design, with a single randomised-control trial. Cohorts were mostly of cirrhosis, but non-cirrhotic chronic hepatitis B (HBV) was also represented. Study periods spanned from 1985 to 2020. Similarly, the characteristics, dimensions of HCC care, axes of health inequality, key findings, and implications of the reports of new studies are presented in Table 2.

**Table 2:** Summary of reports of new included studies addressing health inequalities in HCC, ordered by year of publication (most recent first).

Study	Design	Cohort and Setting	Period	Dimensions of Care	Axes of Health Inequality	Findings	Implications
Ramai et al., 2023 [52]	Systematic review and meta-analysis	4550 patients across 9 studies (5 USA, 2 Australia, 1 UK, 1 Thailand)	2013–2020	Surveillance	Accessing services	Interventional programmes increase uptake. Clinical reminders had 4 times higher adherence.	Implementation of locally tailored surveillance programs. Patient/community navigators may be useful. Low quality of evidence. Randomised controlled trial of different surveillance interventions is recommended.
Wolf et al., 2021 [53]	Systematic review and meta-analysis Mostly cohort studies	118,799 patients across 29 studies (18, USA, 6 Europe, 2 Asia, 1 UK, 1 Canada)	1994–2017	Surveillance	Accessing services Aetiology Age Ethnicity Sex Region	Pooled estimate for surveillance utilization of 24.0% (95%CI 18.4–30.1). Adherence associated with subspecialist f/u and interventional surveillance (8 studies). Poor adherence in ARLD/MASLD.	Interventions included patient/provider education, in-reach (e.g. reminder and recall), population health out-reach.
Zhao et al., 2018 [54]	Systematic review and meta-analysis	19,511 patients across 22 studies (13 USA, 7 Europe, 1 UK, 1 Asia)	1985–2015	Surveillance	Accessing services Aetiology Region	Pooled adherence rate was 52% (39% in retrospective studies). Adherence associated with cirrhosis > HBV, semi-annual interval, combination ultrasound + AFP, and prospective design.	Prospective studies considered less real-world. Heterogeneity a problem. Better understanding of barriers from provider and patient perspective.

*HCC* hepatocellular carcinoma, *USA* United States of America, *UK* United Kingdom, *CI* confidence interval, *ARLD* alcohol related liver disease, *MASLD* metabolic dysfunction-associated steatotic liver disease, *HBV* hepatitis B virus, *AFP* alpha-fetoprotein.

### Axes of Health Inequality in HCC

The findings are presented as ‘axes of health inequality’, this term has been used to refer to the dimensions of individual identity, status and social position that influence health outcomes. These axes help to identify the marginalised groups that are affected by health inequalities. This concept is closely related to ‘social determinants of health’, which refers to the wider societal conditions that also shape and sustain unequal health outcomes. There are references to the wider literature where appropriate for context.

#### Age

There is an inverse relationship as HCC incidence increases with age but access to curative treatment diminishes [13]. Older adults are also more likely to be diagnosed with HCC through emergency presentation and in late stages, with associated reduced survival [12, 13]. In cirrhosis with cured chronic hepatitis C (HCV), increasing age may be associated with better surveillance adherence despite potential reducing benefit [25]. Across the cancer care continuum more broadly, clinical outcomes for older adults are often inferior. Treatment is complicated by the need to adapt to baseline health and performance status, which can vary widely. Higher rates of socio-economic deprivation with advancing age and barriers to accessing care, further compound health inequalities [26].

#### Sex

Higher incidence and mortality in males has been observed longitudinally across the UK [1, 12, 27–31]. This reflects global patterns of disease [32], and is largely explained by clustering of risk factors in men and differences in sex hormones [33]. In addition, hepatic iron levels are a co-factor in fibrosis progression and the relative iron deficiency observed in menstruating women appears protective, although this advantage is lost post-menopause [34].

#### Ethnicity

Asian and Black Caribbean ethnic minority groups are disproportionately affected with higher incidence and mortality [28, 29, 31], this is largely explained by higher viral hepatitis incidence in migrants from endemic countries but genetic differences probably have a role [35]. However, there is also a growing burden of metabolic dysfunction-associated steatotic liver disease (MASLD) across diverse ethnicities worldwide [36]. In terms of the patient journey, higher rates of emergency presentation and late stage diagnosis, and reduced access to curative treatment are observed for ethnic minority groups in the UK [12]. This may be partly due to higher rates of socio-economic deprivation [37] and greater barriers to accessing services such as cancer screening [38].

#### Socioeconomic status

Local socioeconomic deprivation levels are strong predictors of inequalities in health [39] and deprivation has now been linked to liver cancer mortality in the UK [40]. Ranked indices of deprivation have been used as a proxy for income level or socioeconomic status. Increasing deprivation is associated with a higher incidence of HCC and late-stage diagnosis through symptomatic routes [12, 13, 27, 29]. Variations in the HCC disease burden have been observed with higher rates in Greater Manchester and London [13], which are presumed due to a combination of higher deprivation and ethnically diverse urban populations. However, deprivation in rural communities is also well established. The highest incidence and mortality affects men in Scotland [1], which could reflect increased exposure to risk factors such as drug and alcohol use [41]. These associations tell only part of the story, the wider societal context is clearly highly relevant but has not been captured by the evidence included in this review.

#### Lifestyle factors and aetiology

An epidemic of lifestyle related liver disease and subsequent HCC is being driven by behaviours including high alcohol consumption, eating low quality diets, and limited physical inactivity [42, 43]. There is conflicting evidence on surveillance adherence across different CLD aetiologies [44], but particularly poor adherence has been observed in cirrhosis with cured HCV [25]. There is a concern that MASLD is underrepresented in surveillance due to a large burden of undetected disease in the community, despite being associated with developing HCC in the absence of cirrhosis [45]. In addition, ultrasound inadequacy is a growing challenge with the increasing prevalence of people living with obesity [46], and data is awaited on the use of abbreviated MRI as an alternative. Alcohol-related liver disease (ARLD) appears associated with reduced access to treatment [14], which could represent the intersection between ongoing alcohol use and systemic inequities experienced by this group.

#### Access to healthcare services

The geographical provision of Hepatology services across the UK is inequitable. Higher uptake of surveillance appears associated with attending Level 3 Hepatology centres [25]. In contrast, increasing travel time is associated with increased death after listing and reduced likelihood of liver transplantation or recovery [23]; it remains debated where would be the optimum site for an additional UK transplant centre to mitigate this effect. London appears to be an outlier with better access to curative treatment and improved survival [14]. This may be due to improved access to Level 3 centres and a relatively younger population with a greater viral hepatitis predominance. The barriers faced by marginalised groups in accessing HCC care pathways remain underexplored and poorly understood.

### Impact on outcomes across the HCC care continuum

The impact of the identified axes of health inequality on outcomes has been presented across the HCC care continuum, from surveillance and diagnosis to treatment and survival. Findings pertinent to the UK population and healthcare system are explored in the context of the wider literature, including research performed in different healthcare systems and cultures.

#### Surveillance

Surveillance appears poorly targeted, inefficient, and inequitable [24, 25, 47]. This reflects a lack of resources and infrastructure nationally despite evidence demonstrating its effectiveness in improving access to curative treatment and reducing mortality [48, 49]. Poor adherence is undoubtedly undermining these benefits, but there is a lack of good quality data and monitoring in the UK [50, 51]. A limited number of single-centre retrospective studies have reported 19–76% adherence to bi-annual surveillance [24, 25, 47], and appears particularly patchy in people with cirrhosis and cured HCV, 9% across all follow-up [25]. This aligns with meta-analyses reporting 24–52% adherence across studies internationally [52–54]. However, the definition of adherence is heterogenous and measurement often fraught with methodological limitations; the quality of data is low and comprehensive subgroup analyses are missing.

A combination of provider factors, such as doubting effectiveness and inappropriate requesting of tests [24, 47, 50, 51], health system factors including limited capacity and complex pathways [55], and patient factors such as non-attendance and related barriers [47, 56] contribute to poor adherence with surveillance standards. Patient-reported barriers and attitudes have not been explored in a UK population but there are emerging learnings on barriers to cancer screening more widely for underserved and marginalised groups, which could be applied [38]. There can be significant misconceptions, such as surveillance not being necessary in the absence of symptoms or after normal tests [52]. Understanding of the importance of timely surveillance is likely suboptimal and requires improved patient communications [50, 51, 55]. In the USA, a strong predictor of continued surveillance is having multiple visits with a liver specialist, and there appears to be an inverse relationship between ultrasound lead time (difference between the dates it was ordered and subsequently performed) and adherence [57].

In the UK, a radiology-led automatic recall system was trialled with no benefit [24]. More widely, other interventions have been explored including focused patient education [58], and interventional surveillance programmes which employ automatic recall, mail outreach/reminders and pathway navigators [52, 53]. All demonstrate potential to improve adherence. Further efforts to design and evaluate interventional surveillance programmes are required.

#### Diagnosis

HCC incidence is growing in the UK [1, 27, 28, 30] and projected to have the highest average annual increase of all cancers over the next 15 years [2]. A large proportion of cases are diagnosed outside of surveillance (60%) [44], the exact reasons are unknown and require further investigation. However, there is a growing burden of undetected CLD in the general population, which requires co-ordinated efforts to improve screening and diagnosis [43]. Symptomatic presentation of HCC is common via emergency (35.6%), GP referral (31.1%) and two-week wait (11.5%) pathways [13], which are associated with a more advanced stage at diagnosis [12]. The Covid-19 pandemic had a negative impact with higher rates of emergency presentation and larger tumour diameter [59]. One study reported an early detection rate of 25.6% for Barcelona Clinic Liver Cancer stage 0-A [44]. However, there is a lack of available national data on cancer stage, liver function and performance status at diagnosis [14].

The reporting method for imaging remains largely unstandardised across UK centres [50], despite the development of tools such as LI-RADS [60, 61] which could help standardise our approach to management [4]. Liver biopsy and histopathological assessment is increasingly required to verify diagnosis in favour of reliance on radiological evidence [28]. This shift may improve access to clinical trials, experimental treatment options and personalised therapy, an area which has been lacking compared to other cancers [62].

#### Treatment and survival

According to a single study with data encompassing 2001–2007, access to curative treatment (ablation, resection, transplantation) appears limited (24.4%), with the majority of patients receiving no treatment at all (58.4%), and only a small proportion undergoing transplantation at any stage (5.5%) [14]. Therapeutic advances such as loco-regional and systemic therapies over the last couple of decades have resulted in only modest improvements and 5-year survival remains low (18.3%) [1]. Intersecting axes of inequality including increasing age and deprivation, ARLD, and black Caribbean and Asian ethnic groups are associated with reduced access to treatment and survival [12–14]. However, rates of treatment utilisation across subgroups and reasons for non-utilisation have not been established in the UK. In addition, stage-specific survival data are not available from HES or cancer registries due to a lack of granularity. Within cancer more widely, increased travel time is associated with more advanced stage at diagnosis, inappropriate treatment and reduced survival [63]. HCC treatment is centralised at specialist centres, therefore travel time may have a significant role in treatment and outcomes and needs further exploration.

## Discussion

**Fig. 2:**
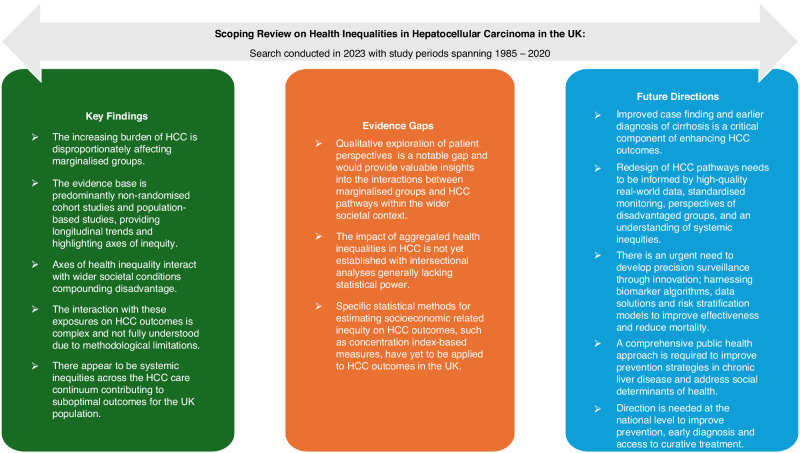
Summary of findings from this scoping reiew on health inequalities in HCC in the UK. A diagram presenting the key findings, evidence gaps and future directions identified. HCC hepatocellular carcinoma, UK United Kingdom.

The discussion provides a synthesis of key findings, appraises the evidence – including its strengths and limitations – and outlines future directions with implications for practice, policy and research, as illustrated in Fig. 2.

### Summary of key findings

This is the first scoping review to characterise axes of health inequality in HCC and their impact across the care continuum in the UK. The incidence and mortality of HCC are increasing and disproportionally affecting marginalised groups who remain underserved by the current healthcare system, presenting a major public health concern. The relationship between exposures and outcomes is complex. There is an interplay between individual axes of health inequality and wider societal conditions, compounded by the increasing burden of liver disease and systemic inequities in HCC care pathways. The fairness of health inequalities is nuanced, acknowledging the distinction of causal variables into ‘circumstances’ beyond individual responsibility (e.g. biological sex and socioeconomic status) and ‘efforts’ for which individuals are responsible (e.g. lifestyle factors) [64]. Regardless, the axes of inequality at play here appear to have a considerable impact with low adherence to HCC surveillance standards, late-stage HCC diagnosis, limited access to curative treatment, and low survival rates common outcomes faced by individuals. Outcomes in the UK are suboptimal in comparison to other leading healthcare systems such as Japan [65]. The axes of health inequality identified in this review have highlighted marginalised groups that experience disproportionately poor HCC outcomes. These findings have underscored the need for targeted consultation and consideration of these groups when redesigning care pathways.

### Appraisal of the evidence

The evidence base is largely comprised of non-randomised cohort studies and observational epidemiological data. This has traditionally been considered a lower quality of evidence. However, it provides an appropriate lens to understand longitudinal trends and identify axes of health inequality in HCC. Several studies have attempted to explore the impact of aggregated axes of health inequality on outcomes through multivariate regression modelling [12, 13, 25, 44, 47]. However, their statistical power was generally insufficient to effectively demonstrate these intersectionalities. Statistical methods for estimating socioeconomic related inequality in health, such as concentration index based measures, have yet to be applied to HCC outcomes in the UK [66]. Qualitative studies exploring patient perspectives are a notable gap at present and would provide valuable insights into the factors that influence interactions between marginalised groups and HCC care pathways within their broader societal context. Furthermore, the meta-analyses on surveillance utilisation predominantly consider studies of non-UK populations, highlighting the paucity of UK data [52–54]. Therefore, differences in ethnic diversity, liver disease aetiology, and healthcare systems (such as a health-insurance model), reduce the transferability of these findings. A scoping review was adopted to map evidence and identify gaps for future research rather than answer a specific question related to HCC care. Therefore, assessing the risk of bias and certainty of evidence were not required.

### Future directions

Improved case finding and earlier diagnosis of cirrhosis in the general population is a critical component of enhancing HCC outcomes. Further research is needed to evaluate the performance of current HCC surveillance and treatment pathways. High-quality data and standardised monitoring are needed to generate real-world evidence that can guide resource allocation to underserved regions and patient groups. Current surveillance strategies fail to account for individual HCC risk or competing outcomes, such as liver decompensation and death. A key priority is to develop robust, risk-based models that enable precision surveillance, enhance cost-effectiveness and reduce mortality [67]. Emerging surveillance tools such as GAAD/GALAD [68–70] and risk stratification models [71–73] move us in the right direction by incorporating age, sex and novel biomarkers within their algorithms. However, significant statistical and clinical challenges remain, and these models require external validation in the UK population before translation into routine practice [74]. To improve HCC care pathways, future efforts must prioritise incorporating the patient perspective and ensuring equitable access across underserved groups. From a policy and practice perspective, a comprehensive public health approach is critical to developing effective prevention strategies for CLD. Organisation and leadership at the national level are required to redesign HCC care pathways, reduce systemic inequities, and meet the urgent need for earlier diagnosis and more equitable access to curative treatments.

## Supplementary information

Supplementary Materials

## Data Availability

No datasets were generated or analysed during the current study.

## References

[1] Burton A, Tataru D, Driver RJ, Bird TG, Huws D, Wallace D, et al. Primary liver cancer in the UK: Incidence, incidence-based mortality, and survival by subtype, sex, and nation. JHEP Rep. 2021;3:100232. 10.1016/j.jhepr.2021.100232 33748727 10.1016/j.jhepr.2021.100232 PMC7966867

[2] Smittenaar CR, Petersen KA, Stewart K, Moitt N. Cancer incidence and mortality projections in the UK until 2035. Br J Cancer. 2016;115:1147–55. 10.1038/bjc.2016.304 27727232 10.1038/bjc.2016.304 PMC5117795

[3] Shelton J, Zotow E, Smith L, Johnson SA, Thomson CS, Ahmad A, et al. 25 year trends in cancer incidence and mortality among adults aged 35-69 years in the UK, 1993-2018: retrospective secondary analysis. BMJ. 2024;384:e076962 10.1136/bmj-2023-076962 38479774 10.1136/bmj-2023-076962 PMC10935512

[4] Suddle A, Reeves H, Hubner R, Marshall A, Rowe I, Tiniakos D, et al. British Society of Gastroenterology guidelines for the management of hepatocellular carcinoma in adults. Gut. 2024;73:1235–68. 38627031 10.1136/gutjnl-2023-331695 PMC11287576

[5] NHS England. Hepatocellular carcinoma: delivering quality ultrasound surveillance 2024 [Available from: https://www.england.nhs.uk/long-read/hepatocellular-carcinoma-delivering-quality-ultrasound-surveillance/#guidance-statements.

[6] Liver Cancer UK. Liver Cancer - A Call to action. British Liver Trust; 2023.

[7] NHS England. Hepatocellular carcinoma surveillance: minimum standards 2024 [Available from: https://www.england.nhs.uk/long-read/hepatocellular-carcinoma-surveillance-minimum-standards/.

[8] Jones PD, Lai JC, Bajaj JS, Kanwal F. Actionable Solutions to Achieve Health Equity in Chronic Liver Disease. Clin Gastroenterol Hepatol. 2023;21:1992–2000. 10.1016/j.cgh.2023.03.043 37061105 10.1016/j.cgh.2023.03.043 PMC10330625

[9] Kondili LA, Lazarus JV, Jepsen P, Murray F, Schattenberg JM, Korenjak M, et al. Inequities in primary liver cancer in Europe: The state of play. J Hepatol. 2024;80:645–60. 10.1016/j.jhep.2023.12.031 38237866 10.1016/j.jhep.2023.12.031

[10] World Health Organization. Health inequalities and their causes 2018 [Available from: www.who.int/news-room/facts-in-pictures/detail/health-inequities-and-their-causes.

[11] Ventura-Cots M, Bataller R, Lazarus JV, Benach J, Pericàs JM. Applying an equity lens to liver health and research in Europe. J Hepatol. 2022;77:1699–710. 10.1016/j.jhep.2022.07.021 35985542 10.1016/j.jhep.2022.07.021

[12] Liao W, Coupland CAC, Innes H, Jepsen P, Matthews PC, Campbell C, et al. Disparities in care and outcomes for primary liver cancer in England during 2008-2018: a cohort study of 8.52 million primary care population using the QResearch database. eClinicalMed. 2023;59. 10.1016/j.eclinm.2023.101969. 10.1016/j.eclinm.2023.101969 PMC10186486 37200996

[13] Burton A, Balachandrakumar VK, Driver RJ, Tataru D, Paley L, Marshall A, et al. Regional variations in hepatocellular carcinoma incidence, routes to diagnosis, treatment and survival in England. Br J Cancer. 2022;126:804–14. 10.1038/s41416-021-01509-4 34837073 10.1038/s41416-021-01509-4 PMC8888669

[14] Beecroft S, O’Connell M, Nassar A, Noon K, Pollock KG, Palmer D, et al. Major variation in hepatocellular carcinoma treatment and outcomes in England: A retrospective cohort study. Frontline Gastroenterol. 2022;14:19–24. 10.1136/flgastro-2022-102142 36561791 10.1136/flgastro-2022-102142 PMC9763635

[15] Marmot M Fair Society, Healthy Lives. 2010 10.1016/j.puhe.2012.05.014 22784581

[16] Alderwick H, Dixon J The NHS long term plan. British Medical Journal Publishing Group; 2019.

[17] NHS England. Core20PLUS5 (adults) – an approach to reducing healthcare inequalities 2021 [Available from: https://www.england.nhs.uk/about/equality/equality-hub/national-healthcare-inequalities-improvement-programme/core20plus5/.

[18] Munn Z, Peters MDJ, Stern C, Tufanaru C, McArthur A, Aromataris E. Systematic review or scoping review? Guidance for authors when choosing between a systematic or scoping review approach. BMC Med Res Methodol. 2018;18:143 10.1186/s12874-018-0611-x 30453902 10.1186/s12874-018-0611-x PMC6245623

[19] Page MJ, McKenzie JE, Bossuyt PM, Boutron I, Hoffmann TC, Mulrow CD, et al. The PRISMA 2020 statement: an updated guideline for reporting systematic reviews. Int J Surg. 2021;88:105906. 33789826 10.1016/j.ijsu.2021.105906

[20] Tricco AC, Lillie E, Zarin W, O’Brien KK, Colquhoun H, Levac D, et al. PRISMA extension for scoping reviews (PRISMA-ScR): checklist and explanation. Ann Intern Med. 2018;169:467–73. 30178033 10.7326/M18-0850

[21] Methley AM, Campbell S, Chew-Graham C, McNally R, Cheraghi-Sohi S. PICO, PICOS and SPIDER: a comparison study of specificity and sensitivity in three search tools for qualitative systematic reviews. BMC Health Serv Res. 2014;14:579 10.1186/s12913-014-0579-0 25413154 10.1186/s12913-014-0579-0 PMC4310146

[22] Haddaway NR, Page MJ, Pritchard CC, McGuinness LA. PRISMA2020: An R package and Shiny app for producing PRISMA 2020-compliant flow diagrams, with interactivity for optimised digital transparency and Open Synthesis. Campbell Syst Rev. 2022;18:e1230. 36911350 10.1002/cl2.1230 PMC8958186

[23] Webb GJ, Hodson J, Chauhan A, O’Grady J, Neuberger JM, Hirschfield GM, et al. Proximity to transplant center and outcome among liver transplant patients. Am J Transpl. 2019;19:208–20. 10.1111/ajt.15004 10.1111/ajt.15004 PMC6491997 29981195

[24] Farrell C, Halpen A, Cross TJ, Richardson PD, Johnson P, Joekes EC. Ultrasound surveillance for hepatocellular carcinoma: service evaluation of a radiology-led recall system in a tertiary-referral centre for liver diseases in the UK. Clin Radio. 2017;72:338.e11–.e17. 10.1016/j.crad.2016.10.019 10.1016/j.crad.2016.10.019 28041651

[25] Hamill V, Gelson W, MacDonald D, Richardson P, Ryder SD, Aldersley M, et al. Delivery of biannual ultrasound surveillance for individuals with cirrhosis and cured hepatitis C in the UK. Liver Int. 2023;43:917–27. 10.1111/liv.15528 36708150 10.1111/liv.15528 PMC10946603

[26] Dharmarajan KV, Presley CJ, Wyld L. Care Disparities Across the Health Care Continuum for Older Adults: Lessons From Multidisciplinary Perspectives. Am Soc Clin Oncol Educ Book. 2021;41:e215–e24. 10.1200/edbk_319841 10.1200/EDBK_319841 33956492

[27] Konfortion J, Coupland VH, Kocher HM, Allum W, Grocock MJ, Jack RH. Time and deprivation trends in incidence of primary liver cancer subtypes in England. J Evaluation Clin Pr. 2014;20:498–504. 10.1111/jep.12188 10.1111/jep.12188 24902884

[28] Ladep NG, Khan SA, Crossey MM, Thillainayagam AV, Taylor-Robinson SD, Toledano MB. Incidence and mortality of primary liver cancer in England and Wales: changing patterns and ethnic variations. World J Gastroenterol. 2014;20:1544–53. 10.3748/wjg.v20.i6.1544 24587630 10.3748/wjg.v20.i6.1544 PMC3925863

[29] Jack RH, Konfortion J, Coupland VH, Kocher HM, Berry DP, Allum W, et al. Primary liver cancer incidence and survival in ethnic groups in England, 2001–2007. Cancer Epidemiol. 2013;37:34–8. 10.1016/j.canep.2012.10.008 23182222 10.1016/j.canep.2012.10.008

[30] West J, Wood H, Logan RF, Quinn M, Aithal GP. Trends in the incidence of primary liver and biliary tract cancers in England and Wales 1971-2001. Br J Cancer. 2006;94:1751–8. 10.1038/sj.bjc.6603127 16736026 10.1038/sj.bjc.6603127 PMC2361300

[31] Haworth EA, Soni Raleigh V, Balarajan R. Cirrhosis and Primary Liver Cancer Amongst First Generation Migrants in England and Wales. Ethnicity Health. 1999;4:93–9. 10.1080/13557859998227 10887465 10.1080/13557859998227

[32] Sung H, Ferlay J, Siegel RL, Laversanne M, Soerjomataram I, Jemal A, et al. Global Cancer Statistics 2020: GLOBOCAN Estimates of Incidence and Mortality Worldwide for 36 Cancers in 185 Countries. CA Cancer J Clin. 2021;71:209–49. 10.3322/caac.21660 33538338 10.3322/caac.21660

[33] Llovet JM, Kelley RK, Villanueva A, Singal AG, Pikarsky E, Roayaie S, et al. Hepatocellular carcinoma. Nat Rev Dis Prim. 2021;7:6 10.1038/s41572-020-00240-3 33479224 10.1038/s41572-020-00240-3 PMC13537433

[34] Rigamonti C, Andorno S, Maduli E, Capelli F, Boldorini R, Sartori M. Gender and liver fibrosis in chronic hepatitis: the role of iron status. Alimentary Pharm therapeutics. 2005;21:1445–51. 10.1111/j.1365-2036.2005.02517.x 15948811

[35] Chavda V, Zajac KK, Gunn JL, Balar P, Khadela A, Vaghela D, et al. Ethnic differences in hepatocellular carcinoma prevalence and therapeutic outcomes. Cancer Reports. 2023;6 (no pagination) 10.1002/cnr2.1821. 10.1002/cnr2.1821 PMC10440848 37344125

[36] Yip TCF, Vilar‐Gomez E, Petta S, Yilmaz Y, Wong GLH, Adams LA, et al. Geographical similarity and differences in the burden and genetic predisposition of NAFLD. Hepatology. 2023;77:1404–27. 10.1002/hep.32774 36062393 10.1002/hep.32774

[37] Ministry of Housing Communities & Local Government. People living in deprived neighbourhoods 2020 [Available from: https://www.ethnicity-facts-figures.service.gov.uk/uk-population-by-ethnicity/demographics/people-living-in-deprived-neighbourhoods/latest/#data-sources.

[38] Okolie C, Hookway A, Wale A, Everitt J, Shaw H, Lewis R, et al. A rapid review of barriers and facilitators to cancer screening uptake (breast, cervical and bowel) in underserved populations. medRxiv. 2022:2022.08.11.22278362 10.1101/2022.08.11.22278362.

[39] Marmot M. Health equity in England: the Marmot review 10 years on. BMJ. 2020:m693 10.1136/bmj.m693. 10.1136/bmj.m693 32094110

[40] Rashid T, Bennett JE, Muller DC, Cross AJ, Pearson-Stuttard J, Asaria P, et al. Mortality from leading cancers in districts of England from 2002 to 2019: a population-based, spatiotemporal study. Lancet Oncol. 2024;25:86–98. 10.1016/S1470-2045(23)00530-2 38096890 10.1016/S1470-2045(23)00530-2 PMC7615518

[41] Curran C, Stanley AJ, Barclay ST, Priest M, Graham J. The association between deprivation and the incidence and survival of patients with hepatocellular carcinoma in the West of Scotland. Expert Rev Gastroenterol Hepatol. 2021;15:1427–33. 10.1080/17474124.2021.1997586 34689659 10.1080/17474124.2021.1997586

[42] Parkin DM, Boyd L, Walker LC. 16. The fraction of cancer attributable to lifestyle and environmental factors in the UK in 2010. Br J Cancer. 2011;105:S77–81. 10.1038/bjc.2011.489 22158327 10.1038/bjc.2011.489 PMC3252065

[43] Williams R, Aspinall R, Bellis M, Camps-Walsh G, Cramp M, Dhawan A, et al. Addressing liver disease in the UK: a blueprint for attaining excellence in health care and reducing premature mortality from lifestyle issues of excess consumption of alcohol, obesity, and viral hepatitis. Lancet. 2014;384:1953–97. 10.1016/S0140-6736(14)61838-9 25433429 10.1016/S0140-6736(14)61838-9

[44] Haq MI, Drake TM, Goh TL, Ahmed A, Forrest E, Barclay S, et al. Effect of Hepatocellular Carcinoma Surveillance Programmes on Overall Survival in a Mixed Cirrhotic UK Population: A Prospective, Longitudinal Cohort Study. J Clin Med. 2021;10:2770. 34202593 10.3390/jcm10132770 PMC8269358

[45] Tan DJH, Ng CH, Lin SY, Pan XH, Tay P, Lim WH, et al. Clinical characteristics, surveillance, treatment allocation, and outcomes of non-alcoholic fatty liver disease-related hepatocellular carcinoma: a systematic review and meta-analysis. Lancet Oncol. 2022;23:521–30. 10.1016/s1470-2045(22)00078-x 35255263 10.1016/S1470-2045(22)00078-X PMC9718369

[46] Hydes TJ, Cuthbertson DJ, Palmer DH, Elshaarawy O, Johnson PJ, Fernando R, et al. Ultrasonography in surveillance for hepatocellular carcinoma in patients with non-alcoholic fatty liver disease. Hepatoma Res. 2023;9:12. 10.20517/2394-5079.2022.97

[47] Selvapatt N, House H, Brown A. Hepatocellular Carcinoma Surveillance: Are We Utilizing It? J Clin Gastroenterol. 2016;50:e8–e12. 10.1097/MCG.0000000000000344 26018132 10.1097/MCG.0000000000000344

[48] Singal AG, Pillai A, Tiro J. Early Detection, Curative Treatment, and Survival Rates for Hepatocellular Carcinoma Surveillance in Patients with Cirrhosis: A Meta-analysis. PLOS Med. 2014;11:e1001624. 10.1371/journal.pmed.1001624 24691105 10.1371/journal.pmed.1001624 PMC3972088

[49] van Meer S, de Man RA, Coenraad MJ, Sprengers D, van Nieuwkerk KM, Klümpen HJ, et al. Surveillance for hepatocellular carcinoma is associated with increased survival: Results from a large cohort in the Netherlands. J Hepatol. 2015;63:1156–63. 10.1016/j.jhep.2015.06.012 26100498 10.1016/j.jhep.2015.06.012

[50] Scott RA, Cross TJS, Clarke C, Khan SA, Ryder SD, Franklin J, et al. Outcomes of National Survey of the Practice of Hepatocellular Carcinoma Surveillance. J Hepatocell Carcinoma. 2023;10:725–31. 10.2147/jhc.s403702 37152438 10.2147/JHC.S403702 PMC10155710

[51] Cross TJS, Villanueva A, Shetty S, Wilkes E, Collins P, Adair A, et al. A national survey of the provision of ultrasound surveillance for the detection of hepatocellular carcinoma. Frontline Gastroenterol. 2016;7:82–9. 10.1136/flgastro-2015-100617 28840911 10.1136/flgastro-2015-100617 PMC5369506

[52] Ramai D, Singh J, Chandan S, Tartaglia N, Ambrosi A, Khan SR, et al. Utilization of Hepatocellular Carcinoma Surveillance Programs in Patients with Cirrhosis: A Systematic Review and Meta-Analysis. J Clin Gastroenterol. 2023;57:198–203. 10.1097/MCG.0000000000001668 34999648 10.1097/MCG.0000000000001668

[53] Wolf E, Rich NE, Marrero JA, Parikh ND, Singal AG. Use of Hepatocellular Carcinoma Surveillance in Patients With Cirrhosis: A Systematic Review and Meta‐Analysis. Hepatology. 2021;73:713–25. 10.1002/hep.31309 32383272 10.1002/hep.31309 PMC7648722

[54] Zhao, Jin M, Le RH, Le MH, Chen VL, Jin M, et al. Poor adherence to hepatocellular carcinoma surveillance: A systematic review and meta-analysis of a complex issue. Liver Int. 2018;38:503–14. 10.1111/liv.13555 28834146 10.1111/liv.13555

[55] Ladhani S, Ohri A, Wong RJ. Disparities in Hepatocellular Carcinoma Surveillance: Dissecting the Roles of Patient, Provider, and Health System Factors. J Clin Gastroenterol. 2020;54:218–26. 10.1097/MCG.0000000000001313 31913877 10.1097/MCG.0000000000001313

[56] Singal AG, Tiro JA, Murphy CC, Blackwell JM, Kramer JR, Khan A, et al. Patient-Reported Barriers Are Associated With Receipt of Hepatocellular Carcinoma Surveillance in a Multicenter Cohort of Patients With Cirrhosis. Clin Gastroenterol Hepatol. 2021;19:987–95. 10.1016/j.cgh.2020.06.049. 32629122 10.1016/j.cgh.2020.06.049 PMC7779689

[57] Goldberg DS, Taddei TH, Serper M, Mehta R, Dieperink E, Aytaman A, et al. Identifying barriers to hepatocellular carcinoma surveillance in a national sample of patients with cirrhosis. Hepatology. 2017;65:864–74. 10.1002/hep.28765 27531119 10.1002/hep.28765

[58] Shaw J, Patidar KR, Reuter B, Hajezifar N, Dharel N, Wade JB, et al. Focused Education Increases Hepatocellular Cancer Screening in Patients with Cirrhosis Regardless of Functional Health Literacy. Digestive Dis Sci. 2021;66:2603–9. 10.1007/s10620-020-06583-x 10.1007/s10620-020-06583-x PMC7933309 32889600

[59] Geh D, Watson R, Sen G, French JJ, Hammond J, Turner P, et al. COVID-19 and liver cancer: lost patients and larger tumours. BMJ Open Gastroenterol. 2022;9:e000794. 10.1136/bmjgast-2021-000794 35450934 10.1136/bmjgast-2021-000794 PMC9023844

[60] American College of Radiology. LI-RADS® US Surveillance v2024 Core2024. Available from: https://www.acr.org/-/media/ACR/Files/RADS/LI-RADS/LI-RADS-US-Surveillance-v2024-Core.pdf.

[61] American College of Radiology. CT/MRI LI-RADS® v2018 CORE2018. Available from: https://www.acr.org/-/media/ACR/Files/RADS/LI-RADS/LI-RADS-2018-Core.pdf.

[62] Llovet JM, Zucman-Rossi J, Pikarsky E, Sangro B, Schwartz M, Sherman M, et al. Hepatocellular carcinoma. Nat Rev Dis Prim. 2016;2:16018. 10.1038/nrdp.2016.18 27158749 10.1038/nrdp.2016.18

[63] Ambroggi M, Biasini C, Giovane CD, Fornari F, Cavanna L. Distance as a barrier to cancer diagnosis and treatment: Review of the literature. Oncologist. 2015;20:1378–85. 10.1634/theoncologist.2015-0110 26512045 10.1634/theoncologist.2015-0110 PMC4679078

[64] Fleurbaey M, Schokkaert E. Unfair inequalities in health and health care. J Health Econ. 2009;28:73–90. 10.1016/j.jhealeco.2008.07.016 18829124 10.1016/j.jhealeco.2008.07.016

[65] Kudo M. Management of Hepatocellular Carcinoma in Japan as a World-Leading Model. Liver Cancer. 2018;7:134–47. 10.1159/000484619 29888204 10.1159/000484619 PMC5985410

[66] Erreygers G, Van Ourti T. Measuring socioeconomic inequality in health, health care and health financing by means of rank-dependent indices: a recipe for good practice. J Health Econ. 2011;30:685–94. 10.1016/j.jhealeco.2011.04.004 21683462 10.1016/j.jhealeco.2011.04.004 PMC3158909

[67] European Association for the Study of the Liver. Policy Statement Risk-based surveillance for hepatocellular carcinoma among patients with cirrhosis 2023 [Available from: https://easl.eu/wp-content/uploads/2023/04/Policy-Statement-Liver-Cancer-Screening_VFF.pdf.

[68] Chan H, Vogel A, Berg T, De Toni E, Kudo M, Trojan J, et al. A comparative analysis of Elecsys GALAD and Elecsys GAAD score to detect early-stage hepatocellular carcinoma in an international cohort. J Hepatol. 2022;77:S937. 10.1016/S0168-8278(22)02154-7

[69] Tayob N, Kanwal F, Alsarraj A, Hernaez R, El-Serag HB. The Performance of AFP, AFP-3, DCP as Biomarkers for Detection of Hepatocellular Carcinoma (HCC): A Phase 3 Biomarker Study in the United States. Clin Gastroenterol Hepatol. 2023;21:415–23. 10.1016/j.cgh.2022.01.047. 35124267 10.1016/j.cgh.2022.01.047 PMC9346092

[70] Singal AG, Tayob N, Mehta A, Marrero JA, El‐Serag H, Jin Q, et al. GALAD demonstrates high sensitivity for HCC surveillance in a cohort of patients with cirrhosis. Hepatology. 2022;75:541–9. 10.1002/hep.32185 34618932 10.1002/hep.32185 PMC8844059

[71] Fan R, Papatheodoridis G, Sun J, Innes H, Toyoda H, Xie Q, et al. aMAP risk score predicts hepatocellular carcinoma development in patients with chronic hepatitis. J Hepatol. 2020;73:1368–78. 10.1016/j.jhep.2020.07.025 32707225 10.1016/j.jhep.2020.07.025

[72] El-Serag H, Kanwal F, Ning J, Powell H, Khaderi S, Singal AG, et al. Serum biomarker signature is predictive of the risk of hepatocellular cancer in patients with cirrhosis. Gut. 2024. 10.1136/gutjnl-2024-332034. 10.1136/gutjnl-2024-332034 PMC11327383 38365278

[73] Kanwal F, Khaderi S, Singal AG, Marrero JA, Asrani SK, Amos CI, et al. Risk Stratification Model for Hepatocellular Cancer in Patients With Cirrhosis. Clin Gastroenterol Hepatol. 2023;21:3296–304.e3. 10.1016/j.cgh.2023.04.019. 10.1016/j.cgh.2023.04.019 PMC10661677 37390101

[74] Innes H, Nahon P. Statistical perspectives on using hepatocellular carcinoma risk models to inform surveillance decisions. J Hepatol. 2023;79:1332–7. 10.1016/j.jhep.2023.05.005 37210001 10.1016/j.jhep.2023.05.005

