# Supplementary material for: Health inequalities in hepatocellular carcinoma surveillance, diagnosis, treatment, and survival in the United Kingdom: a scoping review
Source: BJC Rep. 2025 Mar 3;3:13. doi: 10.1038/s44276-025-00126-5 (PMC11876667; doi:10.1038/s44276-025-00126-5)
Supplement: Supplementary file 1 — Supplementary Materials [file 44276_2025_126_MOESM1_ESM.docx]

## Supplementary Materials

### Comprehensive Search Strategy

**Health Inequalities in HCC**

1. inequit* OR equit* OR inequal* OR dispar* OR variat* OR differen* OR utili* OR adherence OR barrier*
2. surveillance OR screen* OR diagnos* OR incidence OR treatment* OR outcome* OR surviv* OR mortality
3. 'hepatocellular carcinoma' OR hcc OR 'liver cancer' OR PLC OR 'liver cell cancer'
4. 1 AND 2 AND 3

### Eligibility Criteria

**Inclusion criteria:**

- Focus on hepatocellular carcinoma or primary liver cancer
- Health inequalities characterised or addressed
- Involves a UK population, defined as studies where the study population included individuals from the UK, even if it also comprised individuals from other countries (e.g., in meta-analyses)
- Article, review, survey
- Up to September 2023

**Exclusion criteria:**

- No involvement of UK population
- Focus on liver disease more broadly
- Abstracts only

## Plain English Summary

**Background to the research**

The most common type of liver cancer is called hepatocellular carcinoma (HCC). The main cause of HCC is underlying liver damage known as cirrhosis. In the United Kingdom (UK), more than 3,400 people are diagnosed with HCC each year. However, due to increasing rates of cirrhosis in the general population, HCC is one of the fastest growing causes of cancer death. There are no symptoms in the early stages of HCC when it can be cured. Therefore, people with cirrhosis are offered regular checks for liver cancer twice per year, known as surveillance. Despite this, HCC is often diagnosed late and few patients receive treatment which may result in being cured. There appear to be unfair and avoidable differences for patients with HCC across the population and between different groups within society. These differences are known as health inequalities and can influence things like access to tests and treatment and how long people are likely to live. There has been no summary of the studies which have looked at this problem for the UK population.

**Aims of the research**

We need a clearer understanding of how health inequalities affect people with HCC in the UK. This knowledge will help shape future research and improve patient care. The long-term goal is to make outcomes from HCC more fair across the population.

**Design and methods used**

We brought together a group of experienced researchers to conduct a thorough review of existing research on this topic, known as a ‘scoping review’. This method was chosen to summarise what we know about how health inequalities influence HCC care and outcomes in the UK. It also describes the type of research that has been done and whether it was suitable to improve our understanding, this helps to identify gaps in our knowledge. Online libraries of scientific articles were searched up to September 2023. From 1,264 articles that were identified, we carefully selected all relevant articles that have been verified by the scientific community. This resulted in 19 articles being included, these were carefully read in full and analysed including assessing the quality of research and its limitations. We then reported the type of research that has been done and the main findings. Finally, we discuss what the findings mean for patients and healthcare in the UK in the context of wider knowledge, including research that has been done in other healthcare systems around the world.

**Key Findings**

We found that HCC is a deadly cancer which is difficult to diagnose and treat early. In the UK, only 1 in 4 patients receive curative treatment and less than 1 in 5 patients are alive at 5 years. Whereas in Japan 3 in 5 patients receive curative treatment and half of patients are alive at 5 years. Japan is considered a world leader in liver cancer care, so this comparison demonstrates there is significant potential for improvement. The combination of low-quality surveillance testing, late stage diagnosis, and limited access to curative treatment appear to be driving poor outcomes in the UK. Health inequalities mean that disadvantaged groups of patients are less likely to have regular testing and more likely to be diagnosed late with reduced survival. The groups that are associated with worse outcomes include older adults, males, people from Asian or Black Caribbean ethnic minorities, people living in deprived areas, or people with alcohol-related liver disease. However, more research is needed to fully understand variations in surveillance quality and the journey from diagnosis to receiving treatment across different patient groups. The thoughts and feelings of patients have not yet been explored, which is an important type of research that will help guide future improvements to care pathways. New strategies for detecting cirrhosis, improving access to surveillance testing and tailoring care to individual patient risk are promising but need further research and development.

**Patient and public involvement**

We have worked with two Patient Advisors, who have personal experience of cirrhosis and HCC, to co-develop this plain English summary. This will help to communicate the findings to the public, healthcare leaders and government policy makers to maximise the impact.

**Dissemination**

We have published this scoping review in a scientific journal to communicate the findings to liver healthcare workers and the wider scientific community. The findings will be presented at conferences about liver disease and liver cancer to share the learnings. This knowledge is directly relevant to the UK population and leaders in healthcare and government, who have a social responsibility to level up health inequalities. Looking forward, it is clear a combination of more research and better organisation and leadership at the national level are required to improve care and outcomes for patients with HCC in the UK.

We have reviewed the research to understand how axes of health inequality impact on health outcomes in primary liver cancer in the UK (accepted by BJC Reports). We co-developed a plain English summary with patient advisors to disseminate to the public, NHS leaders and government policy makers to gain traction.
